# Co-Analysis of Transcriptome and Metabolome Reveals Flavonoid Biosynthesis in *Macadamia Pericarp* Across Developmental Stages

**DOI:** 10.3390/foods14213618

**Published:** 2025-10-23

**Authors:** Liang Tao, Qingyi Long, Jinyan Chen, Qin Zhang, Guangzheng Guo, Fengping He, Hu Cai, Jianjian Geng, Ximei Song, Hui Zeng, Wenlin Wang, Fan Yang, Zhuanmiao Kang, Xinghao Tu

**Affiliations:** 1 Yunnan Institute of Tropical Crops, Jinghong 666100, China; 2 Guizhou Institute of Subtropical Crops, Guiyang 550025, China; 3 Key Laboratory of Tropical Fruit Biology, Ministry of Agriculture & Rural Affairs, South Subtropical Crops Research Institute, Chinese Academy of Tropical Agricultural Sciences, Zhanjiang 524091, China; 4 Guangxi South Subtropical Agricultural Science Research Institute, Longzhou 532415, China; 5 Institute of Tropical and Subtropical Cash Crops, Yunnan Academy of Agricultural Sciences, Ruili 678600, China

**Keywords:** *Macadamia integrifolia*, flavonoids, gene expression, metabolic regulation

## Abstract

The pericarp of *Macadamia integrifolia* represents a promising but underexplored source of functional flavonoids. To systematically elucidate their biosynthesis and enhance the industrial potential of this by-product, we conducted integrated transcriptomic and metabolomic profiling of pericarps across five developmental stages (50, 80, 110, 140, and 170 days after flowering). Our analysis reveals, for the first time, a distinct temporal shift in both gene expression and metabolite accumulation. Early stages were characterized by high expression of PAL, *4CL*, *CHS*, and *FLS*, coupled with abundant flavonols and anthocyanins. In contrast, late stages exhibited upregulation of *CHI* and *F3’5’H*, redirecting the metabolic flux toward flavanones and isoflavones. This dynamic profile was closely associated with jasmonate and gibberellin signaling pathways and was likely regulated by key transcription factors (MYB, NAC, bHLH). These findings provide a multi-omics framework that elucidates the temporal flavonoid biosynthesis in macadamia pericarp, thereby laying the groundwork for its future industrial valorization.

## 1. Introduction

*M. integrifolia* is a perennial woody oil-bearing and food crop species belonging to the Proteaceae family. Its kernel exhibits a crisp texture and rich flavor after drying, along with high nutritional value. Macadamia represents a significant economic crop in tropical regions of China. In recent years, the macadamia industry has developed rapidly in Chinese provinces such as Yunnan, Guangxi, and Guizhou. The macadamia fruit primarily comprises three parts: the husk (exocarp), the shell (woody endocarp), and the kernel. The husk (exocarp) develops from the tissue differentiation of the ovary wall. As the green fleshy structure encasing the macadamia shell, the husk constitutes approximately 50% of the average fruit weight and represents the principal by-product generated during processing [1]. Current research on macadamia fruit predominantly focuses on the processing and utilization of the kernel and shell, while studies investigating the composition and potential applications of the husk remain scarce [2].

Flavonoids, which belong to the group of phenolic compounds, are a class of important and widely distributed secondary metabolites in plants. They can be classified into several subclasses, primarily including flavones, flavonols, flavanones, flavanols (also known as flavan-3-ols), isoflavones, and anthocyanins [3]. Abundant in vegetables and fruits, these compounds possess various bioactivities, including antiviral [4], antioxidant [5], and anti-inflammatory effects [6]. Research indicates that flavonoids constitute the primary functional components in plant pericarps. Metabolomic analysis of phenolic acids in macadamia (*Macadamia* spp.) revealed variations in the types and concentrations of phenolic acid metabolites produced in the pericarp at different developmental stages [7]. The pericarp of purple passion fruit (*Passiflora edulis* Sims) contains structurally diverse flavonoids, specifically including flavones, flavonols, and anthocyanins, with flavonoid glycosides, encompassing both C-glycosides and O-glycosides, being the most abundant [8]. In melon (*Cucumis melo* L.), the polyphenol content in pericarp extracts was found to be higher than that in seeds [9]. To date, over 300 distinct compounds have been identified in species belonging to the Proteaceae family. The chemical profile is dominated by three major classes: phenolic compounds (69%), quinones (8%), and alkaloids (13%) [10,11]. Metabolomic analysis of macadamia kernels and pericarps revealed that amino acids and their derivatives were the most abundant class of differentially accumulated metabolites. The relative content of amino acids in the pericarp was 1.34 times that in the kernel. Furthermore, flavonoids accumulated significantly higher in the pericarp, with a level 3.14 times that of the kernel, and represented the second most numerous class of differential metabolites between the kernel and pericarp [12].

As a major class of plant secondary metabolites, flavonoid biosynthesis is regulated by multiple factors. Studies have shown that abscisic acid (ABA), ethylene (ETH), jasmonates (JA), cytokinins (CTKs), and brassinosteroids (BRs) promote flavonoid biosynthesis, whereas auxin (IAA) acts as a negative regulator [13]. The ability of phytohormones to modulate flavonoid biosynthesis is evident across species. For example, ABA upregulation of genes like *VvPAL*, *VvCHS1*, *VvF3’H*, *VvF3’5’H*, and *VvDFR* promotes anthocyanin production in grape berries [14]. Similar regulatory roles have been reported for ETH in red pear flavonoid synthesis [15] and for preharvest methyl jasmonate application in enhancing raspberry flavonoid content [16]. In apple, the IAA repressor protein *MdIAA26* mediates auxin-induced suppression of anthocyanin accumulation and downregulates anthocyanin biosynthetic genes in callus, leaves, and seedlings [17]. It was found that the contents of anthocyanins, as well as cyanidin-3-O-glucoside and cyanidin-3-O-rutinoside, were significantly elevated by the application of exogenous ABA in fig (*Ficus carica* L.) fruits [18]. Ethylene promotes flavonoid biosynthesis in red pear fruit [15]. Transcription factors, which bind specifically to promoter regions of target genes, play crucial roles in regulating plant growth and development. Members of the MYB, bHLH, WRKY, NAC, and bZIP families are key regulators of flavonoid biosynthesis [19,20,21].

Current research on macadamia pericarp has primarily focused on the extraction and identification of compounds, while the biosynthetic mechanism of flavonoids in this tissue remains largely unexplored. With increasing fruit production, the processing phase generates substantial quantities of pericarp. Analyzing the functional flavonoid components in macadamia pericarp, extracting, and utilizing these compounds can significantly enhance economic value, optimize resource utilization, and reduce environmental burdens. This study collected pericarp samples from macadamia at various post-anthesis stages for transcriptomic and targeted metabolomic analyses. The objective is to investigate the functional components of flavonoids within the macadamia pericarp and elucidate their biosynthetic mechanisms, thereby providing a foundation for the subsequent development and utilization of these functional constituents.

## 2. Materials and Methods

### 2.1. Plant Materials and Treatment

The plant material used in this study was the pericarp of macadamia nuts, with the selected cultivar being ‘Nanya 116’, an excellent variety independently bred in China. The experimental samples were collected from Xingyi City, Guizhou Province, China (104°59′36″ E, 24°52′46″ N), at an altitude of approximately 780 m. Pericarps from five distinct developmental stages were selected for analysis: 50, 80, 110, 140, and 170 days after flowering. Healthy, mature macadamia trees with uniform vigor were selected. From these, five undamaged fruits of uniform size and maturity were randomly sampled from each of the upper, middle, and lower canopy positions per tree to constitute one biological replicate, with three such replicates being analyzed for every developmental stage. The pericarps were carefully excised from the fruits and immediately flash-frozen in liquid nitrogen. All samples were subsequently stored at −80 °C until further analysis for transcriptome sequencing, targeted metabolomic profiling, and determination of flavonoid content.

### 2.2. Metabolite Profiling and Data Analysis

The non-targeted metabolomics of macadamia seedling leaves was commissioned by the Wekemo Tech Group Co., Ltd. (Shenzhen, China). Data acquisition was carried out using ultra-performance liquid chromatography (UPLC) coupled with tandem mass spectrometry (MS/MS). After the test is completed, the relevant data are normalized by normalizing the total peak area. Metabolite identification was achieved by referencing a self-constructed metabolite database. The relative content of each metabolite was represented by the integrated peak area of product ions within the corresponding chromatographic retention time. Peak area data for all detected spectral features were exported to obtain both qualitative and quantitative metabolite results. Differentially abundant metabolites (DAMs) were screened according to the criteria of |fold change| > 2, *p*-value < 0.05, and variable importance in projection (VIP) > 1 [22,23]. Functional annotation of the identified metabolites was carried out using the KEGG (https://www.genome.jp/kegg/pathway.html (accessed on 28 April 2025)), HMDB (https://hmdb.ca/metabolites (accessed on 28 April 2025)), and LIPIDMaps (http://www.lipidmaps.org/ (accessed on 28 April 2025)) databases.

### 2.3. Transcriptome Analysis

The processes transcriptome sequencing was all completed by the Wekemo Tech Group Co., Ltd. and the sequencing analysis is completed based on Illumina HiSeq sequencing platform. Reference genome was downloaded from the NCBI database [24]. The transcriptomic and metabolomic analyses were performed on identical biological samples. Differential expression analysis of two groups was performed using the DESeq2 R package (1.16.1). The resulting *p*-values were adjusted using the Benjamini and Hochberg’s approach for controlling the false discovery rate. Genes with an adjusted *p*-value < 0.05 found by DESeq2 were assigned as differentially expressed. Functional enrichment analyses of GO terms and KEGG pathways for the DEGs were conducted using the clusterProfiler package (version 3.4.4).

### 2.4. Quantitative Real-Time PCR

To independently verify the transcriptomic results, the expression levels of six candidate genes were assessed by qRT-PCR. This analysis utilized the same RNA samples as the transcriptome sequencing. Gene-specific primers were designed with Primer 5.0 (see Table S1 for sequences), and the entire qRT-PCR protocol, including experimental steps and calculation methods, was conducted in accordance with the work of Li et al. [25].

### 2.5. Statistical Analysis

Microsoft Excel 2010 was employed for data processing. Statistical significance, evaluated by one-way ANOVA followed by Duncan’s multiple range test (*p* ≤ 0.05), was analyzed using SPSS 26.0. All graphical representations were created in GraphPad Prism 9.4.1 (La Jolla, CA, USA).

## 3. Results

### 3.1. Changes in Flavonoid Content in the Pericarp of Macadamia

**Figure 1:**
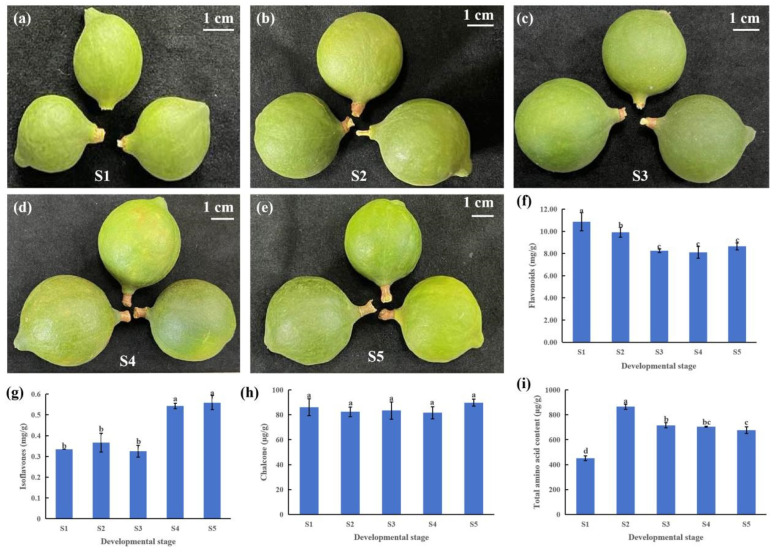
Phenotypic and physiological changes during macadamia pericarp development. (**a**–**e**) Developmental states of macadamia pericarps at different stages: S1, S2, S3, S4, and S5 represent 50, 80, 110, 140, and 170 days after fruit set, respectively; (**f**) total flavonoid content; (**g**) isoflavone content; (**h**) chalcone content; (**i**) total amino acid content. Different lowercase letters indicate significant differences in the developmental stages (*p* < 0.05).

To investigate the changes in flavonoid compounds during the development of macadamia pericarp, we collected pericarps at 50, 80, 110, 140, and 170 days after fruit set. The developmental states of the pericarps at each stage are shown in Figure 1a–e. The contents of total flavonoids, isoflavones, chalcones, and total amino acids in the pericarps at different stages were determined using high-performance liquid chromatography (HPLC). Total flavonoids represent the sum of all flavonoid compounds, serving as an important indicator of the overall content of these bioactive compounds in plants. In macadamia pericarp, the total flavonoid content showed no significant difference between stages S1 and S2, although it was higher in S1 than in S2. The content in S3, S4, and S5 was lower than that in S1 and S2, with no significant differences among these three stages. The content in the macadamia nut peel at stage S2 is significantly lower than that at stage S1. Overall, the flavonoid content in macadamia pericarp decreased initially and then increased (Figure 1f). The content of isoflavones generally exhibited an increasing trend, reaching the highest level at the late developmental stage (S5), which was approximately 1.67 times that of S1 (Figure 1g). No significant differences were observed in the chalcone content across the five developmental stages, with values remaining around 80 μg/g (Figure 1h). Previous studies have indicated that amino acids and their derivatives are the most abundant class of differential metabolites detected in both the pericarp and kernel of macadamia. In this study, the total amino acid content was highest in the pericarp at 80 days after flowering, followed by a gradual decreasing trend (Figure 1i).

### 3.2. Metabolomic Analysis of Pericarp at Different Developmental Stages

**Figure 2:**
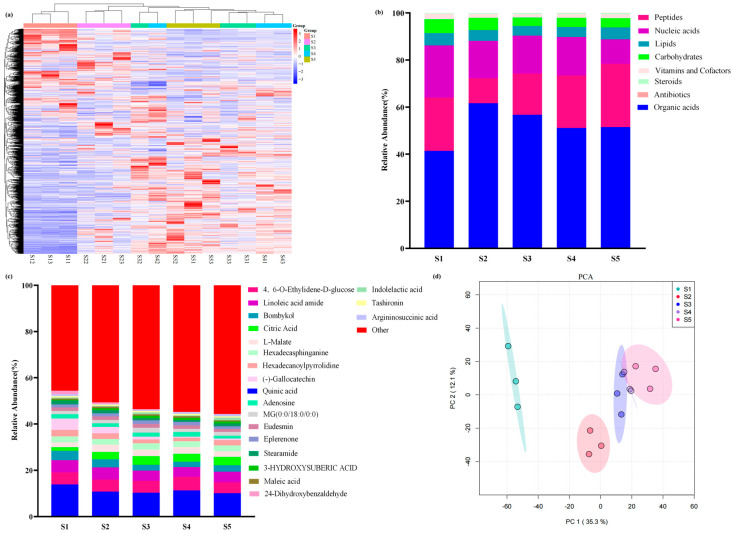
(**a**) Cluster heatmap of metabolomic data from macadamia pericarp at different developmental stages. The labels S11, S12, and S13 denote the first, second, and third biological replicates of sample S1, respectively; S21, S22, and S23 represent the three biological replicates of sample S2. The same nomenclature applies to subsequent samples S3, S4, and S5; (**b**) stacked bar chart of metabolite classification; (**c**) stacked bar chart of the top 20 abundant metabolites in the pericarp; (**d**) principal component analysis (PCA) of the metabolomic data.

We collected the pericarps of macadamia nuts at 50, 80, 110, 140, and 170 days after fruit set for metabolomic analysis. A total of 2749 metabolites were detected across all samples, comprising 87 substances with biological roles and 2662 other compounds (Figure 2a). The metabolites with biological roles were primarily classified into eight major categories: 20 lipids (including 19 polypeptides), 13 steroids, 11 carbohydrates, 9 vitamins and cofactors, 8 organic acids, 6 nucleic acids, and 1 antibiotic (Figure 2b). Statistical analysis of the top 20 most abundant metabolites revealed that the highest levels were mainly quinic acid, 4,6-O-Ethylidene-α-D-glucose, linoleamide, (E,Z)-10,12-Hexadecadienol, citric acid, L-malic acid, catechin, gallocatechin, among others (Figure 2c). In the principal component analysis (PCA), PC1 accounted for 35.3% and PC2 for 12.1% of the variance (For the loadings of each principal component, see Supplementary Table S2), indicating clear separation of macadamia pericarp samples across different developmental stages and confirming the reliability of the metabolomic data (Figure 2d). This dataset was subjected to further analysis.

**Figure 3:**
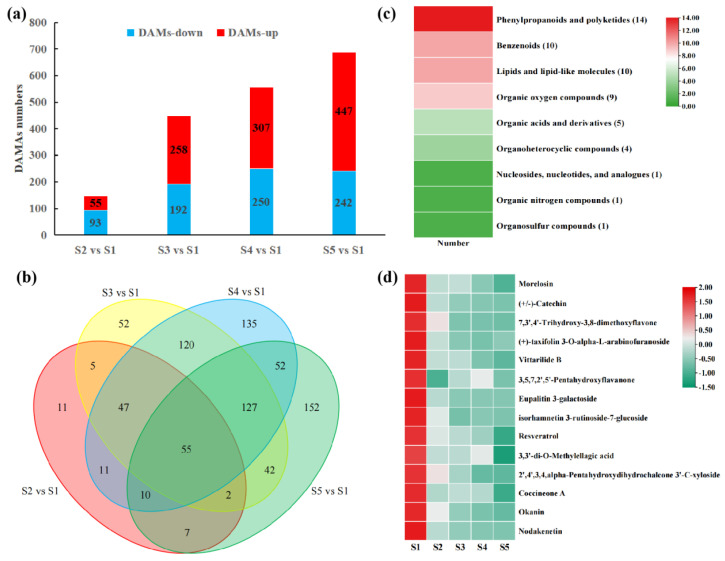
Analysis of pericarp metabolome data at different developmental stages. (**a**) Differential metabolites in the four comparison groups; (**b**) Venn diagram of differential metabolites among the four comparison groups; (**c**) statistical heatmap of the classification of common differential metabolites across comparison groups; (**d**) abundance heatmap of phenylpropanoid and polyketide differential metabolites.

By further utilizing Fold Change and *p*-values to screen for differential metabolites, metabolites satisfying |log_2_(Fold Change)| > 1 and *p* < 0.05 were considered differential. A total of 817 differential metabolites were detected across the comparison groups S2 vs. S1, S3 vs. S1, S4 vs. S1, and S5 vs. S1 (Figure 3a). Among these, 55 differential metabolites were common to all four comparison groups (Figure 3b). These differential metabolites were primarily classified into nine major categories, with phenylpropanoids and polyketides being the most abundant, comprising 14 metabolites (Figure 3c). Further analysis revealed that these metabolites exhibited a decreasing trend during fruit development (Figure 3d).

**Figure 4:**
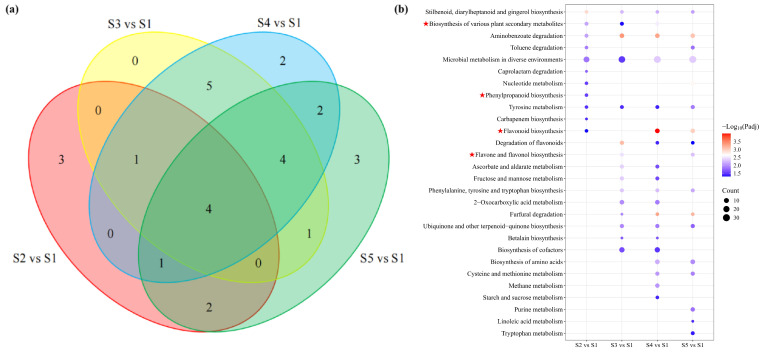
Enrichment analysis of metabolites. (**a**) Venn diagram of KEGG enrichment analysis for differential metabolites; (**b**) bubble plot of KEGG enrichment analysis for differential metabolites.

Based on the KEGG database, differential metabolite accumulation across different developmental stages was explored, with pathways screened using *p* < 0.05 as the criterion. The results revealed that 11, 15, 19, and 19 metabolic pathways were significantly enriched in the S2 vs. S1, S3 vs. S1, S4 vs. S1, and S5 vs. S1 comparison groups, respectively. Among these, 4 pathways were commonly enriched across all four comparison groups (Figure 4a). Analysis of these enriched pathways identified that the differential metabolites were significantly enriched in Microbial metabolism in diverse environments, Aminobenzoate degradation. This study investigates the synthesis of functional components in macadamia pericarp, with flavonoids being widely distributed secondary metabolites in the plant kingdom. In addition to the pathways mentioned above, we also found that the DEGs were enriched in multiple flavonoid-related pathways, such as Phenylpropanoid biosynthesis, Biosynthesis of various secondary metabolites, Flavonoid biosynthesis, and Flavone and flavonol biosynthesis. Further analysis of the flavonoid biosynthesis pathway will be conducted subsequently (Figure 4b).

### 3.3. Transcriptome Analysis of the Pericarp at Different Developmental Stages of Macadamia

**Figure 5:**
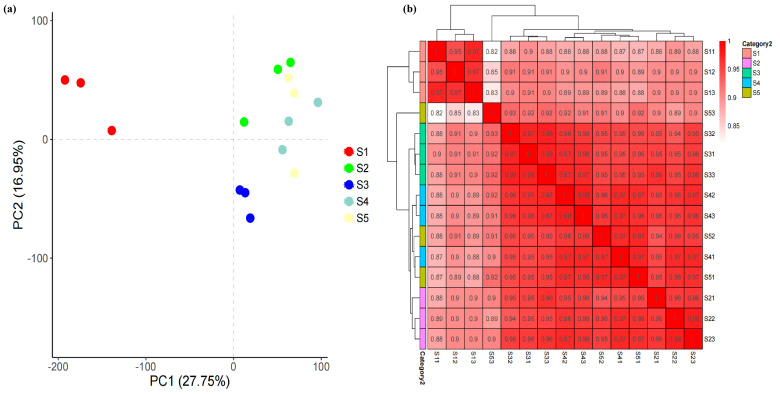
Transcriptome data analysis. (**a**) PCA of the identified genes in the samples. (**b**) Correlation heatmap in all samples. The labels S11, S12, and S13 denote the first, second, and third biological replicates of sample S1, respectively; S21, S22, and S23 represent the three biological replicates of sample S2. The same nomenclature applies to subsequent samples S3, S4, and S5. The labels S11, S12, and S13 denote the first, second, and third biological replicates of sample S1, respectively; S21, S22, and S23 represent the three biological replicates of sample S2. The same nomenclature applies to subsequent samples S3, S4, and S5.

Metabolomic data analysis revealed that the flavonoid biosynthesis pathway significantly influences the synthesis of functional components in macadamia pericarps. We conducted transcriptome profiling of macadamia pericarp across different developmental stages to elucidate the response patterns of these functional components. This process yielded approximately 40.14–64.54 million high-quality clean reads after data filtering and quality control for all subsequent analyses. Within the constructed libraries, the Q20 and Q30 statistics for the Clean Reads were greater than 99.54% and 97.53%, respectively. The Clean Reads from each sample were then mapped to the macadamia reference genome, achieving mapping rates ranging from 88.20% to 89.30% (Table S3). Through this mapping process, 30,520 known genes (79.72% of the total 38,284) were detected, along with 1441 novel genes predicted to be absent from the reference genome. The identification of DEGs was conducted by analyzing variations in expression levels among the samples. These DEGs were subsequently subjected to functional annotation and enrichment analysis. Principal component analysis (PCA) and intra-group correlation analysis were conducted on the transcriptome data from all 15 samples. The PCA results indicated that principal component 1 (PC1) and principal component 2 (PC2) explained 27.75% and 16.95% of the total gene expression variance across all samples, respectively (Figure 5a). Correlation heatmap analysis revealed high correlation coefficients (R^2^ > 0.82) among the biological replicates for each sampling time point (Figure 5b), demonstrating the stability and reliability of the transcriptome data.

### 3.4. Identification and Functional Annotation of DEGs in Pericarp Across Developmental Stages

**Figure 6:**
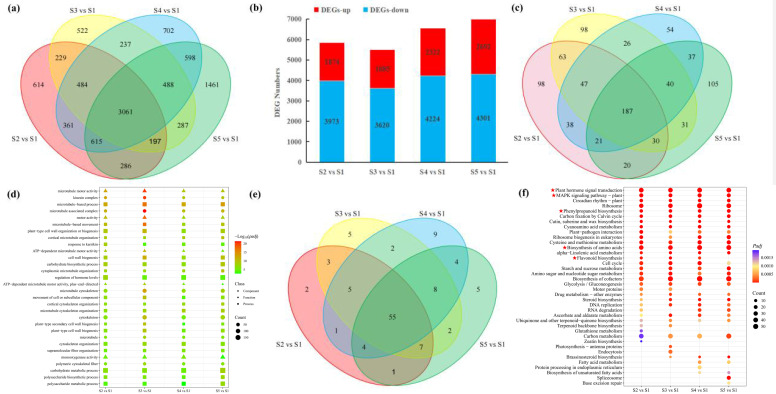
Transcriptome data analysis. (**a**) Venn diagram of differential metabolites in macadamia pericarp across developmental stages; (**b**) Bar plot showing the statistics of differentially expressed genes; (**c**) Venn diagram of GO enrichment analysis; (**d**) Bubble plot of GO enrichment analysis for differentially expressed genes. The GO categories are represented by different shapes: Cellular Component (CC) by a circle, Molecular Function (MF) by a triangle, and Biological Process (BP) by a square. The term “S2 vs. S1” signifies the GO pathways enriched by the DEGs identified from the comparison of stage S2 versus stage S1. The same applies to “S3 vs. S1”, “S4 vs. S1”, and “S5 vs. S1”; (**e**) Venn diagram of KEGG enrichment analysis; (**f**) Bubble plot of KEGG enrichment analysis for differentially expressed genes. The term “S2 vs. S1” signifies the GO pathways enriched by the DEGs identified from the comparison of stage S2 versus stage S1. The same applies to “S3 vs. S1”, “S4 vs. S1”, and “S5 vs. S1”.

To investigate the transcriptional changes during pericarp development in macadamia (*Macadamia integrifolia*), differential gene expression analysis was performed on the obtained genes. A total of 10,637 DEGs were identified across ten comparison groups between different developmental stages (Figure 6a). The pairwise comparisons of S2, S3, S4, and S5 against S1 collectively identified 10,142 DEGs. A detailed distribution showed 5847 DEGs (1874 up-regulated, 3973 down-regulated) in S2 vs. S1; 5505 (1885 up, 3620 down) in S3 vs. S1; 6546 (2332 up, 4224 down) in S4 vs. S1; and 6993 (2692 up, 4301 down) in S5 vs. S1 (Figure 6b). Notably, an intersection of these comparisons revealed a common core of 3061 DEGs. Functional annotation of the DEGs was performed based on the Gene Ontology (GO) database (Figure 6c). Significantly enriched GO terms (*p* < 0.05) numbered 504, 522, 450, and 471 for the S2 vs. S1, S3 vs. S1, S4 vs. S1, and S5 vs. S1 comparisons, respectively, with 187 terms significantly enriched across all four groups. Enriched terms were categorized into three ontologies: Biological Process (BP), Molecular Function (MF), and Cellular Component (CC), with BP containing the highest number of enriched terms. The top 20 most significant GO terms from each comparison group were selected for further analysis. Cumulatively, these represented 34 unique GO terms, the majority of which belonged to BP. These processes are closely associated with fruit development.

Pathway enrichment analysis employing the Kyoto Encyclopedia of Genes and Genomes (KEGG) database was carried out to explore the expression patterns of DEGs throughout developmental stages, using a significance threshold of *p* < 0.05. This revealed 78, 87, 88, and 86 significantly enriched metabolic pathways in the S2 vs. S1, S3 vs. S1, S4 vs. S1, and S5 vs. S1 comparisons, respectively (Figure 6d). Fifty-five pathways were commonly enriched across all four comparison groups (Figure 6e). The identified metabolites were significantly enriched in several key metabolic pathways, including plant hormone signal transduction, amino acid biosynthesis, phenylpropanoid biosynthesis, flavonoid biosynthesis, MAPK signaling pathway–plant, circadian rhythm–plant, ribosome, Calvin cycle, carbon fixation, as well as cutin, suberine, and wax biosynthesis (Figure 6f).

**Figure 7:**
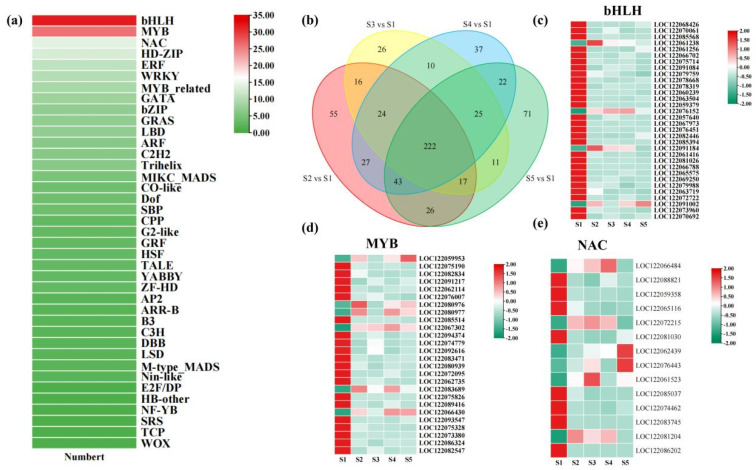
Analysis of transcription factors in macadamia pericarp across different developmental stages. (**a**) Heatmap of common differentially expressed transcription factors among the four comparison groups; (**b**) Venn diagram of differentially expressed transcription factors in the four comparison groups; (**c**) expression heatmap of bHLH transcription factors; (**d**) Expression heatmap of MYB transcription factors; (**e**) expression heatmap of NAC transcription factors.

Plant transcription factors (TFs) are key regulators governing essential processes such as growth and development, morphogenesis, and adaptation to environmental stimuli. To pinpoint pivotal TFs associated with pericarp development in macadamia, we systematically examined the TF repertoire derived from transcriptome data. Our analysis revealed a total of 632 differentially expressed TFs throughout five developmental stages of macadamia (Figure 7a). Among these, 222 TFs exhibited significant differential expression within the four comparison groups (Figure 7b). Remarkably, the bHLH, MYB, and NAC families constituted the most abundantly represented TF groups, comprising 32, 26, and 14 members, respectively, indicating their potential importance in regulating pericarp development in macadamia (Figure 7c–e).

### 3.5. Integrated Analysis of Transcriptome and Metabolome Profiles Across Different Developmental Stages of Macadamia Pericarp

**Figure 8:**
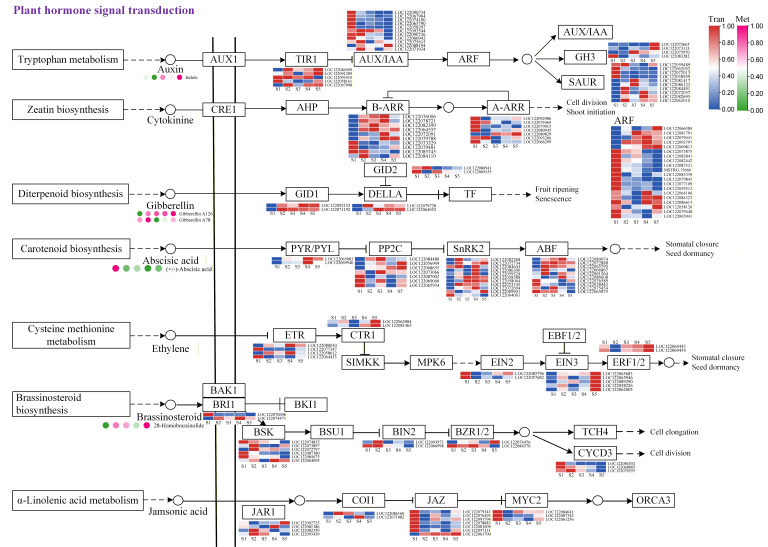
Expression Profiles of Key Genes in Plant Hormone Signal Transduction Pathways.

Previous studies have established that flavonoid biosynthesis is regulated by plant hormones. Consistent with this, our transcriptomic analysis revealed that a substantial number of DEGs identified during macadamia pericarp development were enriched in plant hormone signal transduction pathways. We subsequently visualized the expression profiles of genes associated with these pathways (Figure 8). The results indicated that within the auxin signaling pathway, the auxin receptor TIR was constitutively expressed throughout all pericarp developmental stages. Several *ARFs* (auxin response factors) exhibited peak expression at the S1 stage. In contrast, two *GH3* (Gretchen Hagen 3) genes, which participate in the negative feedback regulation of auxin homeostasis, were predominantly expressed at the S5 stage. Additionally, several *SAUR* (Small Auxin-Up RNA) genes were highly expressed during the S1 and S2 stages, with lower expression in later stages compared to the early stages. The overall change in indole, an important precursor of IAA synthesis, showed a gradual increase in relative content as the fruit developed. In the CTK signal transduction pathway, multiple *B-ARR* (Type-B Arabidopsis Response Regulator) genes, which encode core transcription factors responsible for activating downstream gene transcription, exhibited high transcript levels during the middle stages of pericarp development (S2, S3, S4). In contrast, the negative feedback regulator *A-ARR* (Type-A Arabidopsis Response Regulator) showed relatively higher expression at the S1 stage. In the GA signal transduction pathway, the relative expression of the gibberellin receptor *GID1* (Gibberellin Insensitive Dwarf 1) gradually increased across the five developmental stages. The expression pattern of *DELLA* a core negative regulator in the GA pathway, was similar to that of *GID1*. *GID2* (Gibberellin Insensitive Dwarf 2), which inhibits the function of *DELLA*, was encoded by a gene highly expressed in the early stages of pericarp development but decreased in later stages. The accumulation pattern of Gibberellin A126 gradually increased during pericarp development, while the relative content of Gibberellin A78 fluctuated, peaking at the S2 stage, decreasing thereafter, and increasing again at the S5 stage. In the ABA signal transduction pathway, genes encoding ABA receptors *PYL/PYR* (PYR1-LIKE/PYRABACTIN RESISTANCE 1) showed higher transcript levels at the S4 and S5 stages of pericarp development. *PP2C* (Protein Phosphatase 2C), a core negative regulator that continuously suppresses downstream signaling in the absence of ABA, was encoded by genes with varying expression patterns across different developmental stages. When *PP2C* activity is inhibited, *SnRK2* (SNF1-related protein kinase 2) activates downstream kinases, serving as a central node in ABA signal transduction. Genes encoding this protein were generally more highly expressed during the later stages of macadamia pericarp development, particularly at the S4 stage. Multiple *ABF* (ABA-responsive element Binding Factor) genes exhibited high transcript levels except at the S1 stage. Meanwhile, the accumulation level of *ABA* overall showed a decreasing trend in the pericarp. In the ethylene signal transduction pathway, *CTR1* (Constitutive Triple Response 1), *EIN3* (Ethylene Insensitive 3), and *ERF1/2* (Ethylene Response Factor 1/2) displayed high transcript levels at the S5 stage, while *EIN2* was highly transcribed at the early S1 stage of pericarp development. In the BRs signal transduction pathway, genes encoding *BIN2* (Brassinosteroid-Insensitive 2) and *CYCD3* (Cyclin D3) proteins showed high transcript levels during the early stages (S1 and S2) of macadamia pericarp development, with lower expression at the S5 stage. High brassinosteroid levels accumulated relatively more at the S2, S3, and S5 stages. In the JA pathway, *JAZ (Jasmonate ZIM-domain)* and *MYC2* were highly transcribed at the S1 stage, while *JAR1* and *COI1* (Coronatine Insensitive 1) showed relatively high expression during the middle stages of pericarp development. In summary, the gene expression patterns in plant hormone signal transduction pathways varied during macadamia pericarp development, indicating their distinct roles in pericarp growth and flavonoid accumulation.

**Figure 9:**
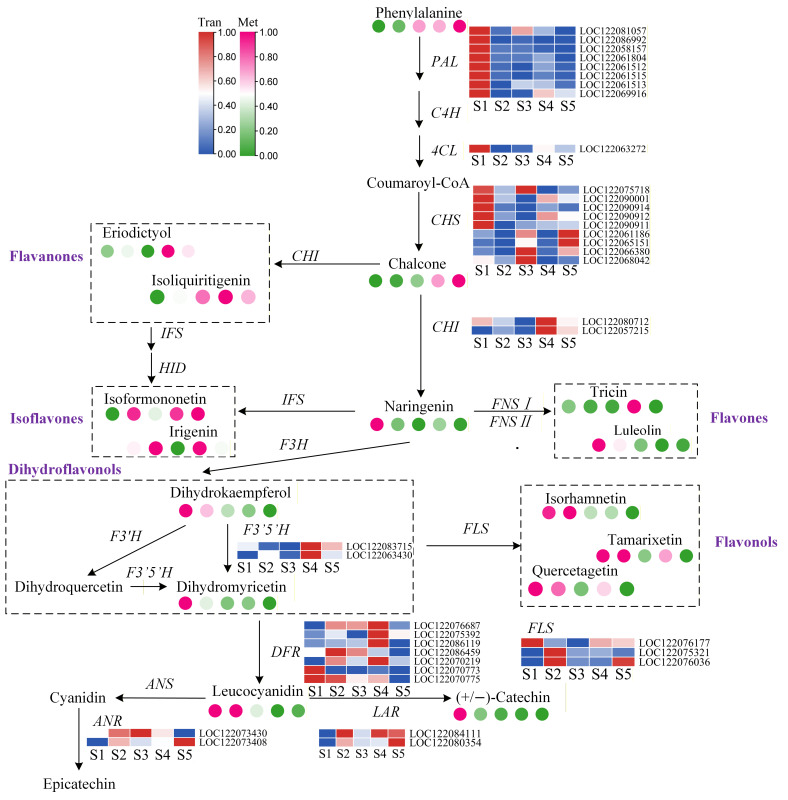
Key Gene Expression and Metabolite Accumulation in the Flavonoid Pathway.

Transcriptomic analysis revealed that DEGs across different developmental stages of macadamia pericarp were significantly enriched in the flavonoid biosynthesis pathway, while the associated metabolites predominantly belonged to phenylpropanoids and polyketides. The flavonoid pathway and the phenylpropanoid pathway are two closely interconnected core pathways in plant secondary metabolism, with the flavonoid pathway being a major branch of phenylpropanoid metabolism. We focused on the flavonoid pathway, a source of common plant functional compounds, and delineated its biosynthetic scheme (Figure 9). A gradual increase in phenylalanine accumulation was observed during pericarp development. In contrast, the transcriptional levels of upstream enzymes, including *PAL* (phenylalanine ammonia-lyase) and *4CL* (4-coumarate:CoA ligase), were highest at the S1 stage. Elevated expression of *CHS* (chalcone synthase), the first key enzyme dedicated to flavonoid formation, was also prominent in early development. Meanwhile, the downstream compound chalcone notably accumulated to higher levels at the S4 and S5 stages. *CHI* (Chalcone isomerase) exhibited higher transcript levels in the later stages (S4 and S5) of pericarp development, and *F3’5’H* (Flavonoid 3’,5’-Hydroxylase) showed a similar expression pattern with elevated expression in the later stages. *DFR* (Dihydroflavonol 4-reductase) and *ANR* (Anthocyanidin reductase) were more highly expressed during the middle stages (S2, S3, S4), while *LAR* (Leucoanthocyanidin reductase) expression was higher in the later stages compared to the initial stage. Analysis of the relative accumulation of flavonoid compounds in macadamia pericarp at different developmental stages revealed that eriodictyol and isoliquirtigenin (flavanones) accumulated more at S4 and S5. Isoformononetin and irifenin (isoflavones) showed considerable variation but higher accumulation at S5. Naringenin accumulation was highest at S1 and subsequently decreased. Dihydrokaempferol and dihydromyricetin (dihydroflavonols) were more abundant at S1 and gradually decreased in later stages. Several downstream flavonols, such as isorhamnetin, tamarixetin, and quercetagetin, also showed higher accumulation in the early stages and lower accumulation in the later stages of pericarp development. The *LAR* and *ANR* genes, which catalyze the synthesis of proanthocyanidins, were more highly expressed in the later stages of pericarp development. In contrast, leucocyanidin and (+/−)-catechin accumulated relatively more in the early developmental stages. In summary, during macadamia pericarp development, the expression levels of genes in the flavonoid biosynthesis pathway and the accumulation of related metabolites exhibited distinct patterns. Upstream genes such as *PAL*, *4CL*, and *CHS* showed high transcript levels in the early stages of pericarp development, while downstream genes including *CHI*, *F3’5’H*, and *LAR* had higher expression in the later stages. In the early stages, flavonols, dihydroquercetin, and downstream anthocyanin compounds accumulated abundantly, whereas in the later stages, flavanones and isoflavones were more predominant. This aligns with physiological data showing higher isoflavone content in the late stages of pericarp development.

### 3.6. Correlation of Flavonoid Biosynthesis and Plant Hormone Signal Transduction Pathway

To investigate whether the development of flavonoids in the pericarp of macadamia nuts interacts with plant hormone signaling transduction pathways, we conducted a Pearson correlation analysis based on key flavonoid compounds, structural genes, and genes involved in plant hormone signal transduction pathways (Table S4). First, we analyzed the metabolites and structural genes in the flavonoid biosynthesis pathway. The upstream *PAL* genes (LOC122086992, LOC122058157, LOC122061804, LOC122061512, LOC122061515) showed highly significant positive correlations with most flavonoid compounds. *DFR* genes (LOC122070219, LOC122070773) were also significantly correlated with (+/−)-catechin, dihydrokaempferol, eriodictyol, isoliquiritigenin, and luteolin. *CHI* (LOC122090914) exhibited highly significant positive correlations with (+/−)-catechin, dihydrokaempferol, and luteolin. These genes also displayed high degree values in the correlation network, suggesting their potential crucial roles in the synthesis of flavonoids in the macadamia pericarp. Subsequently, we performed a correlation analysis between the metabolites and genes of the flavonoid biosynthesis pathway and the top thirty most abundant genes in the plant hormone signal transduction pathway based on Pearson correlation coefficients (Table S5). The results revealed that most JA signaling pathway genes, including *JAZ* and *MYC2*, showed highly significant correlations with metabolites and genes involved in flavonoid biosynthesis. Specifically, they were significantly positively correlated with early-accumulating compounds such as (+/−)-catechin, dihydrokaempferol, luteolin, and naringenin chalcone, while exhibiting highly significant negative correlations with late-accumulating compounds like isoformononetin and isoliquiritigenin. *JAZ* and *MYC2* were also significantly positively correlated with multiple *PAL* genes, as well as with *DFR* (LOC122086459) and *CHI* (LOC122090911). Additionally, ARF and *GID1* showed highly significant correlations with metabolites and genes in the flavonoid biosynthesis pathway. These findings suggest a mutual interaction between flavonoid biosynthesis and plant hormones.

Validation was performed by subjecting key genes from the flavonoid biosynthesis pathway to qRT-PCR analysis (Figure S1). The resulting expression data corroborated the transcriptomic findings, thereby validating the reliability of the RNA-seq dataset and justifying its application in subsequent correlation analyses.

## 4. Discussion

### 4.1. The Important Role of Flavonoids in Macadamia Pericarp Development

*Macadamia*, one of the four major tree nuts, is rich in essential nutrients. With the expansion of the macadamia industry, the large volume of processing by-products generated during production has led to resource wastage and environmental pollution. Flavonoids are widely occurring phenolic compounds in plants with diverse functions. On one hand, they contribute to plant color formation [26], auxin transport [27], and defense against pathogen infection [28,29]. On the other hand, flavonoids are beneficial to human health; for example, quercetin and catechin exhibit anti-inflammatory effects on macrophages [30], while quercetin and rutin demonstrate antitumor activity [31]. Previous studies have shown that among the downregulated metabolites in macadamia kernel and pericarp, medicarpin-5-O-hexoside, (-)-gallocatechin, and proanthocyanidins exhibited the largest fold changes, with downregulation factors of 8.85, 8.45, and 8.18, respectively—all of which belong to the flavonoid class [13]. In this study, we used transcriptome sequencing to analyze gene expression levels in pericarps at different developmental stages (S1–S5). Except for the S5 vs. S1 comparison, DEGs in all other comparative groups were significantly enriched in the flavonoid biosynthesis pathway. Measurements of flavonoid content in pericarps across stages revealed a gradual increase in isoflavones as development progressed. Research on the dynamic development of macadamia fruits in Guizhou indicated that rapid growth in fruit weight, fruit diameter, and nut weight occurred within the first 60 days after flowering [32]. In this study, the changes in total flavonoids and chalcones during pericarp development were not pronounced, which may be attributed to the relatively stable phase of dynamic fruit development after 50 days post-flowering, resulting in no significant fluctuations. Metabolomic studies of the pericarp and kernel identified a large number of DAMs belonging to amino acids and their derivatives [13]. Here, we measured the total amino acid content in the pericarp and found that it peaked at the S2 stage, showing an overall trend of initial increase followed by a decrease, albeit with minor fluctuations. This suggests that amino acids may support pericarp development as nutritional components. Studies have shown that amino acids are essential for the growth and development of most plants. Additionally, metabolomic data analysis detected a substantial number of phenylpropanoids and polyketides. Analysis of the expression patterns of genes involved in the flavonoid biosynthesis pathway and the accumulation of corresponding metabolites revealed distinct patterns for different compounds in macadamia pericarp. Overall, both gene expression and metabolite accumulation varied throughout pericarp development. Upstream genes such as *PAL*, *4CL*, and *CHS* showed high transcript levels in the early stages of pericarp development, providing a foundation for the synthesis of various flavonoids in later stages. The FLS gene was also highly expressed during S1 and S2. In contrast, downstream genes including *CHI*, *F3’5’H*, and *LAR* exhibited higher expression in the later stages of macadamia development. These genes are key structural genes in the flavonoid biosynthesis pathway and play crucial roles in the biosynthesis of flavonoids in the pericarp. Different transcriptional patterns of *CHI* can induce the synthesis of different flavonoids [33]. Dihydroflavonols and flavonols accumulated at higher levels in the early stages of pericarp development, whereas flavanones and isoflavones were more abundant in later stages. This may be because chalcones are increasingly channeled into the flavanone and isoflavone branches under the influence of *CHI* in later phases, leading to higher accumulation of metabolites in these two branches. While we have identified various flavonoid compounds in the macadamia pericarp, we lack absolute quantification of these individual constituents. Precisely determining the predominant flavonoids and their accumulation dynamics is a prerequisite for their subsequent industrial extraction and utilization.

### 4.2. The Influence of Plant Hormones on Flavonoid Biosynthesis in Macadamia Pericarp

Phytohormones are trace organic compounds synthesized by plants themselves. They can be transported from their production sites to target tissues, where they exert critical regulatory effects on plant growth, development, physiological processes, and responses to environmental stimuli at extremely low concentrations. Studies have shown that phytohormones play regulatory roles in the biosynthesis of plant flavonoids. The biological process of phytohormone signal transduction has been observed to be active during the later stages of tuber color formation [34]. Exogenous application of SA and ABA in blueberries has been demonstrated to enhance the expression of flavonoid-related genes and increase anthocyanin content [35]. It has been established that ABA induces anthocyanin biosynthesis by activating the flavonoid biosynthesis pathway [36,37]. In this study, transcriptome analysis of the macadamia pericarp detected numerous components of the ABA signal transduction pathway, including PYR/PYL, PP2C, SnRK2, and ABF. These genes exhibited high transcript levels during the middle stages of pericarp development (S2, S3, and S4), while ABA content peaked at the S1 stage, indicating that ABA plays an important role in flavonoid metabolism during pericarp development. *GA3ox1* (Gibberellin 3-beta-dioxygenase 1) is a key enzyme gene in the “final step” or “rate-limiting step” of the GA pathway, responsible for catalyzing the formation of bioactive gibberellins, thereby directly controlling plant growth and development. In *ga3ox1* mutants of *Medicago truncatula*, the levels of naringenin and apigenin were significantly increased [14]. In this study, the expression of genes related to the gibberellin signal transduction pathway gradually increased during pericarp development, and the contents of Gibberellin A126 and Gibberellin A78 also rose with fruit maturation. This trend was consistent with the accumulation patterns of flavanones and isoflavones, suggesting that the content of these flavonoids in the pericarp may be influenced by gibberellins. In plum fruit, ethylene application was found to markedly enhance the transcription of seven key structural genes in the anthocyanin biosynthesis pathway, including *PsPAL*, *PsCHS*, *PsCHI*, *PsF3H* (Flavanone 3-hydroxylase), *PsDFR*, *PsLDOX* (Leucoanthocyanidin dioxygenase), and *PsUFGT* (UDP-glucose:flavonoid 3-O-glucosyltransferase). The study also identified two ethylene receptors (*PsERS1* and *PsETR1*) and seven ethylene response factors (*PsERF*), whose expression patterns showed a positive correlation with *PsMYB10* and the majority of anthocyanin structural genes [38]. Similarly, research conducted on apples demonstrated that the ethylene response factor MdERF1B physically interacts with *MdMYB9*, *MdMYB1*, and *MdMYB11*, leading to a substantial promotion of anthocyanin and proanthocyanidin accumulation in callus cultures [13]. MeJA can significantly induce the expression of *CtCYP82G24* in safflower, and overexpression of this gene increases flavonoid accumulation in transgenic Arabidopsis [17]. MeJA has also been shown to induce flavonoid accumulation in various fruits, including strawberries, grapes, and blueberries [39,40]. In this study, we found that multiple genes in the JA signal transduction pathway were significantly positively correlated with genes and metabolites related to flavonoid biosynthesis. Our findings support the conclusion that phytohormones regulate the biosynthesis of flavonoids in the macadamia pericarp. Although numerous studies have demonstrated that phytohormones affect flavonoid biosynthesis, particularly anthocyanins, some evidence also suggests that flavonoids can modulate the metabolic levels of phytohormones such as IAA, thereby regulating IAA concentration at cellular and tissue levels and influencing growth and development [41]. In this study, correlation analysis has indicated a link between flavonoid biosynthesis and plant hormone signaling in the developing macadamia pericarp. However, the hierarchical order (i.e., which pathway acts upstream or downstream) awaits further elucidation.

### 4.3. The Impact of Transcription Factors on Flavonoid Biosynthesis in Macadamia Pericarp

The biosynthesis of flavonoids in plants is co-regulated by the synergistic interaction between structural genes and regulatory transcription factors. Key structural genes involved in the flavonoid pathway include *PAL*, *C4H*, *CHS*, *CHI*, *F3H*, *FLS*, and *DFR*, all of which are regulated by transcription factors such as MYB, WRKY, bHLH, and NAC [15,18,19]. In various plant species, specific MYB transcription factors have been identified as key activators of the flavonoid pathway. In Pyrus bretschneideri, *PbMYB10b*, *PbMYB9*, and *PbMYB3* upregulate critical structural genes such as *CHS*, *CHI*, *UFGT*, and *FLS* [42,43]. Similarly, Freesia hybrida studies show that *FhMYBFs* and *FhMYB21* activate *FhFLS1* and *FhFLS2* expression, fulfilling distinct regulatory roles across floral tissues during development [18]. This regulatory function is conserved in *Juglans sigillata*, where MYB members enhance flavonoid accumulation by promoting the expression of biosynthesis-related genes [25]. Consistent with this pattern, the durian MYB factor, *DzMYB*, directly regulates flavonoid biosynthesis by binding to the promoters of *CHS*, *F3H*, and *CHI* genes [44]. The bHLH family represents the second largest superfamily of transcription factors in plants after MYB. In 1989, Chandler et al. first identified a bHLH transcription factor involved in the regulation of flavonoid biosynthesis in maize (*Zea mays*) [45]. The promotory role of bHLH transcription factors in flavonoid biosynthesis has since been extensively demonstrated in various plant species, including narcissus (*Narcissus tazetta*) [46], spine grape (*Vitis davidii*) [47], and barrel medic (*Medicago truncatula*) [48]. bHLH proteins facilitate flavonoid accumulation either by directly interacting with biosynthetic genes or by forming complexes with other transcription factors. For instance, in onion (*Allium* cepa), AcB2 interacts with *AcMYB1* and enhances its transcriptional activity, thereby increasing the expression of *ANS* and *F3H* genes and inducing anthocyanin synthesis [49]. In snapdragon (*Antirrhinum majus*), the *AmDEL* gene promotes the expression of *CHS*, *F3H*, *DFR*, and *FLS* in transgenic plants, leading to enhanced flavonoid accumulation and consequently improved salt tolerance and drought resistance [50]. In the transcriptomic data of macadamia pericarp development, a substantial number of transcription factors were annotated, predominantly belonging to the bHLH, MYB, and NAC families. These transcription factors generally exhibited higher transcript levels during the early stages of pericarp development, suggesting their potential involvement in the biosynthesis of flavonoids throughout this process. To elucidate the regulatory roles of these transcription factors, further functional characterization is necessary to identify the key regulators in macadamia pericarp development and to uncover how they facilitate flavonoid accumulation in the pericarp.

## 5. Conclusions

*M. integrifolia* is a nutrient-dense nut valued for its high-quality lipids and proteins, yet its main by-product—the pericarp—remains underexplored. This study provides the first integrated transcriptomic and metabolomic analysis of the macadamia pericarp across developmental stages, revealing its substantial potential as a source of natural antioxidants and bioactive flavonoids. We identified that phenylpropanoids and polyketides dominated the metabolomic profile, with DEGs significantly enriched in both flavonoid biosynthesis and plant hormone signaling pathways. Functional analysis further highlighted key genes (*PAL*, *CHI*, *FLS*, *DFR*) regulating flavonoid composition: flavonols and anthocyanins accumulated predominantly in the early stages, whereas flavanones and isoflavones increased later. Hormonal signals—JA, GA, and ABA—were strongly correlated with these shifts, and transcription factors (MYB, NAC, bHLH) showed stage-specific expression, suggesting a multi-level regulatory network. These findings not only elucidate the dynamic and stage-specific nature of flavonoid biosynthesis in macadamia pericarp but also establish a new conceptual framework for by-product valorization. For future work, we propose: (1) functional validation of candidate genes and transcription factors via transgenic systems; (2) optimization of extraction protocols for flavanones and isoflavones from mature pericarp. This study provides a theoretical foundation for understanding the biosynthesis of flavonoids in macadamia pericarp, which will facilitate the future extraction and utilization of functional compounds from pericarp by-products in macadamia production.

## Data Availability

The original contributions presented in this study are included in the article. Further inquiries can be directed to the corresponding authors.

## Supplementary Materials

The following supporting information can be downloaded at: https://www.mdpi.com/article/10.3390/foods14213618/s1, Figure S1: qRT-PCR validation of the transcriptome data results for 6 selected genes. The blue folds represent FPKM values in transcriptome data, and the vertical bars shown in the columns represent relative expression level by qRT-PCR. Relative expression levels of qRT-PCR were calculated using 18sRNA as a standard. Select fruit samples from different developmental stages of the pericarp for qRT-PCR analysis, and set the normalized gene level of S1 arbitrarily to 1. Pearson correlation coefficients were calculated by comparing qRT-PCR and FPKM for each gene.; Table S1: Transcriptome data statistics table of 15 samples of macadamia nut peels; Table S2: Sequences of primers used in qRT-PCR; Table S3: Pearson correlation analysis among important flavonoids with important structural genes in the flavonol synthesis pathway; Table S4: The Pearson correlation between plant hormone signal transduction pathways and the flavonoid pathway.
