# Supplementary material for: Co-Analysis of Transcriptome and Metabolome Reveals Flavonoid Biosynthesis in Macadamia Pericarp Across Developmental Stages
Source: Foods. 2025 Oct 23;14(21):3618. doi: 10.3390/foods14213618 (PMC12607494; doi:10.3390/foods14213618)
Supplement: Supplementary file 1 [file foods-14-03618-s001.zip › Figure S1.pdf]

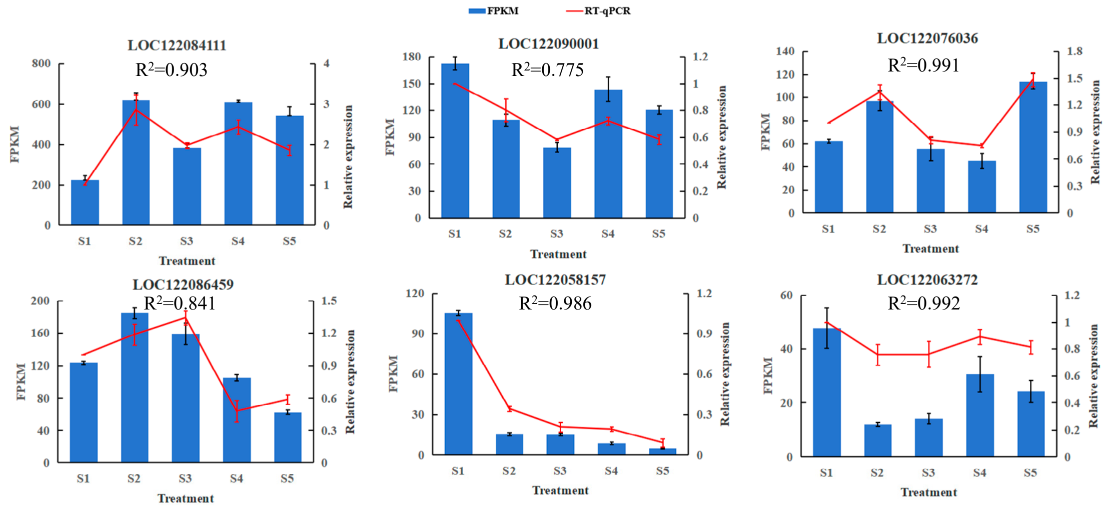

Figure S1. qRT-PCR validation of the transcriptome data results for 6 selected genes. The blue folds represent FPKM values in transcriptome data, and the vertical bars shown in the columns represent relative expression level by qRT-PCR. Relative expression levels of qRT-PCR were calculated using 18sRNA as a standard. Select fruit samples from different developmental stages of the pericarp for qRT-PCR analysis, and set the normalized gene level of S1 arbitrarily to 1. Pearson correlation coefficients were calculated by comparing qRT-PCR and FPKM for each gene.
